# P04.63. The consciousness of medical doctors about collaborative practice of Western medicine and traditional Korean medicine

**DOI:** 10.1186/1472-6882-12-S1-P333

**Published:** 2012-06-12

**Authors:** J Ryu, Y Yun, B Lim

**Affiliations:** 1 Pusan National University School of Korean Medicine, Yangsan, Gyeongnam, Republic of Korea

## Purpose

This study was designed to compare consciousness about collaborative practice between Western medicine (WM) and traditional Korean medicine (TKM) of conventional medical doctors who work in conventional hospitals and those who work in collaborative institutions of WM and TKM (collaborative institution) and to provide policy implication for the development of collaborative practice.

## Methods

Structured questionnaires were mailed to 132 doctors working in non-collaborative practicing university hospitals and 77 doctors working in a collaborative institution in Busan metropolitan city. The response rate was 40.2% and 40.3% respectively. This survey was performed from 10 Oct. 2008 to 31 Oct. 2008.

## Results

Doctors working in conventional hospitals had comparatively negative consciousness regarding the basic concept, remedial value and necessity for collaborative practice and TKM. In regards with disease treatment’s effectiveness of collaborative practice, both groups evaluated musculoskeletal and immune disease were more effective than others. There were positive relationships between perception for cost-effectiveness and consciousness about intention to participate collaborative practice (p<0.05). Also, doctors who experienced TKM treatment had positive consciousness about collaborative practice (p=0.05).

## Conclusion

To activate the collaborative practice of WM and TKM, some efforts should be carried out. These include promoting cooperative education programs in medical schools and traditional Korean medical schools, doing research on cost- effectiveness of collaborative practice, and trying to minimize legal and systemic restrictions for collaborative practice.

